# Livestock-associated meticillin-resistant *Staphylococcus aureus* (MRSA) among human MRSA isolates, European Union/European Economic Area countries, 2013

**DOI:** 10.2807/1560-7917.ES.2017.22.44.16-00696

**Published:** 2017-11-02

**Authors:** Pete Kinross, Andreas Petersen, Robert Skov, Evelyn Van Hauwermeiren, Annalisa Pantosti, Frédéric Laurent, Andreas Voss, Jan Kluytmans, Marc J Struelens, Ole Heuer, Dominique L Monnet, the European human LA-MRSA study group

**Affiliations:** 1 European Centre for Disease Prevention and Control (ECDC), Stockholm, Sweden; 2 Statens Serum Institut, Copenhagen, Denmark; 3 International Center for Infectiology Research (CIRI), INSERM, Lyon, France; 4 Radboud University Medical Centre, Nijmegen, the Netherlands; 5 University Medical Centre Utrecht, Utrecht, the Netherlands; 6 European Programme for Public Health Microbiology (EUPHEM), European Centre for Disease Prevention and Control (ECDC), Stockholm, Sweden; 7 National Institute of Health, Rome, Italy; 8 The members of the group are listed at the end of the article

**Keywords:** cross-sectional studies, livestock-associated, meticillin-resistant Staphylococcus aureus (MRSA) in humans, Europe

## Abstract

Currently, surveillance of livestock-associated meticillin-resistant *Staphylococcus aureus* (LA-MRSA) in humans in Europe is not systematic but mainly event-based. In September 2014, the European Centre for Disease Prevention and Control (ECDC) initiated a questionnaire to collect data on the number of LA-MRSA from human samples (one isolate per patient) from national/regional reference laboratories in European Union/European Economic Area (EU/EEA) countries in 2013. Identification of LA-MRSA as clonal complex (CC) 398 by multilocus sequence typing (MLST) was preferred, although surrogate methods such as *spa*-typing were also accepted. The questionnaire was returned by 28 laboratories in 27 EU/EEA countries. Overall, LA-MRSA represented 3.9% of 13,756 typed MRSA human isolates, but it represented ≥ 10% in five countries (Belgium, Denmark, Spain, the Netherlands and Slovenia). Seven of the reference laboratories did not type MRSA isolates in 2013. To monitor the dispersion of LA-MRSA and facilitate targeted control measures, we advocate periodic systematic surveys or integrated multi-sectorial surveillance.

## Introduction

*Staphylococcus aureus* colonises the anterior nares of nearly all domesticated animals and ca 30% of humans [1-5]. Livestock-associated meticillin-resistant *S. aureus* (LA-MRSA) poses a zoonotic risk, particularly for those working in close contact with livestock [6]. The highest livestock densities in European Union/European Economic Area (EU/EEA) countries in 2013 were in Benelux countries (Belgium, the Netherlands, and Luxembourg), Nordic countries (Denmark and Norway) and Mediterranean islands (Cyprus and Malta) [7]. Several lineages of LA-MRSA have been described [8-10]. The most widespread LA-MRSA lineage in Europe and North America is clonal complex (CC) 398 including multilocus sequence type (MLST) ST398, which is commonly associated with swine, but has also been identified in cattle and poultry [5,8,11]. Carriage of MRSA CC398 is common in individuals with frequent livestock contact, such as swine farmers and people living in areas with high livestock density [2,11]. The impact of this carriage on otherwise healthy persons appears to be low and LA-MRSA infections have a similar severity to that of other MRSA infections [2,11].

In 2014, the death of four individuals from LA-MRSA CC398 in Denmark led to considerable political and media attention in Nordic countries and the European Parliament regarding the potential burden of LA-MRSA in pigs and humans [12]. The risk management options for LA-MRSA include the identification of transmission chains to interrupt transmission. In addition, consistently wearing face masks when working in pig stables has been shown to lower MRSA carriage rates by 37% [13].

Within Decision 2012/506/EU on case definitions for reporting communicable diseases, reporting of MRSA in the EU/EEA is included under the ‘special health issue’ of ‘Antimicrobial resistance’ [14]. The European Centre for Disease Prevention and Control (ECDC) coordinates European surveillance of MRSA through the European Antimicrobial Resistance Surveillance Network (EARS-Net), which collects national clinical laboratory data on invasive MRSA isolates in EU/EEA countries. However, subtyping information that would allow for identification of LA-MRSA isolates is not collected as part of the routine antimicrobial resistance (AMR) surveillance at the European level [15].

A survey conducted in 2006 to 2007 of 357 hospital-serving laboratories in 26 European countries did not identify LA-MRSA among invasive MRSA isolates from humans [3]. In 2007, a survey acquired data on MRSA and LA-MRSA, including screening samples from 21 staphylococcal reference laboratories in 15 countries. Eight countries reported LA-MRSA isolates from humans, including clinical isolates; the proportion of MRSA that were LA-MRSA was above 2% in four countries and in one region in Germany [16].

In 2010, a survey of 29 European countries showed that 19 had a system for surveillance of MRSA, of which 10 had mandatory reporting of MRSA cases [17]. The most frequently used typing methods were DNA sequencing of the repeat region of the *S. aureus* protein A gene (*spa*-typing; n = 25), PFGE (n = 24), staphylococcal cassette chromosome *mec* (SCC*mec*) typing (n = 24), multilocus sequence typing (MLST; n = 20), toxin gene profiling (n = 17) and multiple-loci variable number tandem repeat analysis (MLVA; n = 6) [17,18]. A laboratory-based system ‘Nordic MRSA’ contains comprehensive *spa*, MLST and Pantone-Valentine leukocidin (PVL) data on isolates typed in Denmark, Finland, Iceland, Sweden and Norway from 2009 to 2014 and is updated regularly [19].

The inter-laboratory reproducibility of *spa*-typing in Europe has been long-established [1,4]. In 2010, 24 of 29 European staphylococcal reference laboratories had access to equipment for *spa*-typing [17]. MLST is currently considered one of the gold standards in molecular typing techniques to investigate the evolution of *S. aureus* [20,21]. Whole genome sequencing (WGS) is rapidly replacing MLST for more in-depth study of *S. aureus*, including its evolution [22,23].

In 2011, ECDC recruited 360 laboratories serving 450 hospitals in 26 European countries to assess the feasibility of linking clinical, epidemiological and *spa*-typing data from *S. aureus* blood stream infections during a six-month investigation period. The common view of the participating European staphylococcal reference laboratories was that acquisition of linked clinical, epidemiological and typing data was not feasible due to the differences in national sampling strategies, the paucity of information provided by clinicians on laboratory request forms and the absence of ‘a systematic and consistent identification (through internationally agreed identifiers) of hospitals and diagnostic laboratories’ [17,18]. Therefore, monitoring and assessing the public health threat posed by LA-MRSA in Europe relies on microbiological confirmations, rather than epidemiological case-based surveillance.

Given the sparse information available at the European level on the occurrence of LA-MRSA in humans, ECDC initiated this study to map the identification of LA-MRSA (i.e. CC398 and ‘other’ LA-MRSA) in EU/EEA countries and the MRSA subtyping capacity/availability in EU/EEA national/regional reference laboratories. The study also aimed to describe the detected LA-MRSA according to their site of isolation.

## Methods

### Survey tool

A retrospective survey collected data on LA-MRSA subtypes identified among MRSA isolates from humans by national or regional reference laboratories between 1 January and 31 December 2013. The questionnaire requested data on the first MRSA-positive clinical sample collected from patients in 2013; screening samples (i.e. carriage) were also accepted. ‘All samples’ includes both clinical and screening samples. It then requested both the number of MRSA isolates that were ST398 and also the number that were ‘other LA-MRSA’. As not all reference laboratories perform MLST, the questionnaire included a list of *spa* types considered surrogates for ST398 and also accepted ‘PFGE non-typeable strains (similar to MRSA ST398)’ as such.

The first 12 questions of the questionnaire requested quantitative data within three sections: A. Denominator data (i.e. the catchment population of the laboratory, supplemented by Eurostat mid-2013 population estimates during analysis), the included sample type (i.e. clinical isolates or all isolates) and the microbiological typing method(s) used; B. Numerator data (e.g. total patients from whom a MRSA isolate was received, one isolate per patient during 2013); and C. the type of sample/body site from which the patient’s first positive MRSA isolate was obtained in 2013 (i.e. blood, wound, urine, respiratory tract, skin or mucosa, and other clinical samples including isolates for which the body site was unknown). The questionnaire concluded with section D. containing two free text descriptions of (i) ‘other’ typing method and (ii) the MRSA subtypes considered by respondents to be ‘other LA-MRSA’. Chi-squared tests were used to evaluate differences in the rate of isolation of MRSA between sub-categories of type of sample/body site of the sample.

The questionnaire was a direct update of that of the 2007 survey [16], with only two changes: the addition of the microbiological typing method ‘other’ to section A. and collection of data on ‘other LA-MRSA types’, i.e. distinct from CC398, to sections B. and C.. The updated questionnaire was pilot tested in three countries (Denmark, France and Italy). The list of ST398-surrogate *spa* types was subsequently updated (Box 1) and provided with the revised questionnaire. All three participants in the pilot survey required less than two hours to acquire the necessary data.

Box 1 **List of surrogates for MLST ST398 provided with the revised questionnaire, European Union/European Economic Area, 2013**

*spa* types t011, t034, t108, t567, t571, t588, t753, t898, t899, t1184, t1250^a^, t1254, t1255, t1446, t1451, t1456, t1457, t1580, t1606, t1793^a^, t1928, t2011, t2123, t2330, t2346, t2370, t2383, t2576, t2582, t2974^a^, t3013, t3075, t3275, t3423, t3625, t3933, t4208, t4652, t4872, t5052, t5095, t5706, t6228, t6575, t7880, t8588, t8704, t9345, t9418, t9517, t10055, t10150, t10485, t10890, t11613, t11681, t12313, t12314, t12841, t13885, t13972, t14075, t14080, t14089.

PFGE non-typeable strains similar to ST398.

LA-MRSA: livestock-associated meticillin-resistant *Staphylococcus aureus*; MLST: multilocus sequence type; ST: sequence type.
^a^ Added following the pilot survey.

### Identification of national contact persons

In November 2014, ECDC National Focal Points for Antimicrobial Resistance (AMR) in all 28 EU countries plus Iceland and Norway were invited to designate a primary and alternate contact person with expertise in molecular surveillance of MRSA for public health purposes and with access to data for the survey in their respective countries. The questionnaire was emailed to the nominated contact persons, or to the countries’ National Focal Point for AMR if no contact person was designated.

## Results

### Responses and catchment area

**Figure 1:**
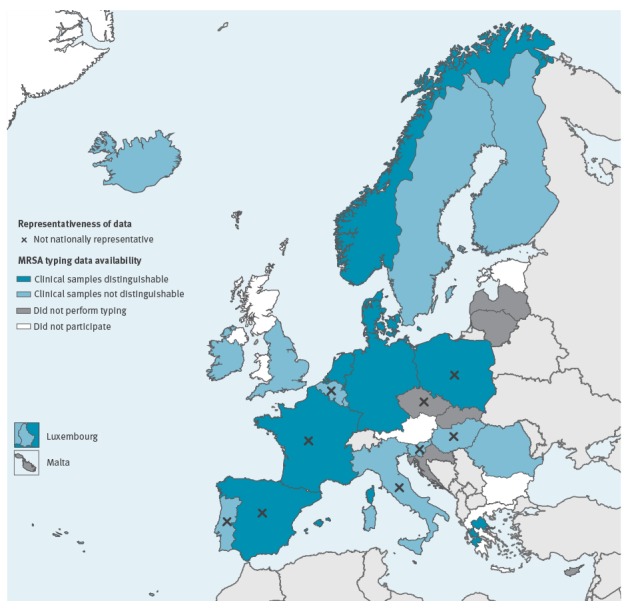
Availability of MRSA typing data in participating national/regional reference laboratory by country, 2013 (n = 27 European Union/European Economic Area countries)

Twenty-eight reference laboratories from 27 of 30 EU/EEA countries responded, 26 of which were national reference laboratories (NRLs) and two were regional reference laboratories. All respondents provided annual data for 2013, except Romania (data only provided for 23 October 2013 to 24 December 2013) (Figure 1).

Administrative boundaries from EuroGeographics. Map produced on 20 October 2017.

MRSA: meticillin-resistant *Staphylococcus aureus*.

Data for the United Kingdom (UK) were only received for England (54 of the 64 million population). Data for Slovenia were received for 1.7 of the 2.1 million population. Two university hospitals in Greece (population 11.1 million) returned data for their respective catchment areas: Thessalia (population 1.2 million) and Patras (population 1.0 million). Even though the questionnaire did not request this information, nine countries (Belgium, Czech Republic, France, Hungary, Italy, Poland, Portugal, Slovenia and Spain) indicated that their data were not nationally representative and/or non-systematic without further quantifying. Several of these respondents did qualify the non-representativeness. For example, in Belgium, laboratories are invited to send outbreak-causing strains to the NRL and are most likely to do so if the epidemiological context is unusual (e.g. including animal contact).

### MRSA typing data availability

Nine laboratories provided data from clinical samples only and 12 laboratories sent aggregated data from both clinical and screening samples. Seven countries (Croatia, Cyprus, Czech Republic, Latvia, Lithuania, Malta and Slovakia) reported that no MRSA typing was performed in 2013, of which five (Croatia, Cyprus, Latvia, Malta and Slovakia) indicated that no typing was performed in their country (Table 1).

**Table 1:** MRSA isolates received and typed by 28 national/regional reference laboratories and the MRSA typing method, 2013 (n = 27 European Union/European Economic Area countries)

Country	Received specimens			MRSA isolates			LA-MRSA isolates				Typing method used			
				Received	Typed		ST398		Other					
	Catchment	Sample types^a^	Period	n	n	%	n	%	n	%	MLST	*spa*	PFGE	Other
Belgium	National^b^	All samples	2013	299	299	100.0	29	9.7	ND	–	Yes (I)	Yes (R)	Yes (I)	Yes (I)^f^
Croatia	National	NA	2013	ND	0	–	NA	–	NA	–	NA	NA	NA	NA
Cyprus	National	NA	2013	ND	0	–	NA	–	NA	–	NA	NA	NA	NA
Czech Republic	National^b^	NA	2013	ND	0	–	NA	–	NA	–	NA	NA	NA	NA
Denmark	National	Clinical only	2013	940	940	100.0	157	16.7	ND	–	No	Yes (R)	No	Yes (R)^g^
Finland	National	All samples	2013	1,330	1,330	100.0	10	0.8	ND	–	Yes (I)	Yes (R)	Yes (I)	No
France	National^b^	Clinical only	2013	300	234	78.0	4	1.7	ND	–	No	No	No	Yes (R)^h^
Germany	National	Clinical only	2013	1,090	1,090	100.0	28	2.6	ND	–	Yes (I)	Yes (R)	No	No
Greece (Patras)	Regional^c^	Clinical only	2013	211	150	71.1	1	0.7	0	0.0	Yes (I)	Yes (I)	No	No
Greece (Thessalia)	Regional^d^	Clinical only	2013	210	193	91.9	0	0.0	0	0.0	Yes (I)	Yes (I)	Yes (R)	No
Hungary	National^b^	All samples	2013	341	191	56.0	12	6.3	0	0.0	Yes (I)	Yes (I)	Yes (R)	No
Iceland	National	All samples	2013	34	34	100.0	0	0.0	0	0.0	No	Yes (R)	No	No
Ireland	National	All samples	2013	556	556	100.0	1	0.2	0	0.0	No	Yes (I)	No	Yes (R)^i^
Italy	National^b^	All samples	2013	42	42	100.0	0	0.0	2	4.8	Yes (I)	Yes (R)	No	No
Latvia	National	NA	2013	ND	0	–	NA	–	NA	–	NA	NA	NA	NA
Lithuania	National	NA	2013	ND	0	–	NA	–	NA	–	NA	NA	NA	NA
Luxembourg	National	All samples	2013	395	395	100.0	16	4.1	0	0.0	Yes (I)	Yes (R)	No	Yes (R)^g,j,k^
Malta	National	NA	2013	ND	0	–	NA	–	NA	–	NA	NA	NA	NA
The Netherlands	National	Clinical only	2013	764	764	100.0	164	21.5	ND	–	No	Yes (R)	No	Yes^i^
Norway	National	Clinical only	2013	659	649	98.5	10	1.5	0	0.0	Yes (I)	Yes (R)	No	No
Poland	National^b^	Clinical only	2013	57	57	100.0	1	1.8	0	0.0	Yes (I)	Yes (R)	Yes (I)	Yes (R)^g^
Portugal	National^b^	All samples	2013	21	21	100.0	0	0.0	0	0.0	Yes (R)	Yes (R)	Yes (I)	Yes (R)^l^
Romania	National	All samples	23 Oct–24 Dec 2013	51	19	37.3	ND	–	ND	–	Yes (I)	Yes (I)	Yes (I)	No
Slovakia	National	NA	2013	ND	0	–	NA	–	NA	–	NA	NA	NA	NA
Slovenia	National^b^	All samples	2013	ND	128	NA	20	15.6	ND	–	Yes (I)	Yes (I)	Yes (I)	No
Spain	National^b^	Clinical only	2013	ND	535	NA	52	9.7	ND	–	Yes (I)	Yes (R)	Yes (R)	No
Sweden	National	All samples	2013	2,454	2,396	97.6	14	0.6	0	0.0	No	Yes (R)	No	No
United Kingdom (England only)	National	All samples	2013	4,537	3,733	82.3	14**^e^**	0.4	0	0.0	Yes (I)	Yes (R)	Yes (I)	Yes (I)^m^
Total	–	–	–	14,291	13,756	96.3	533	3.9	2	0.01	–	–	–	–

–: not calculated; I: on indication; LA-MRSA: livestock-associated meticillin-resistant *Staphylococcus aureus*; MLST: multilocus sequence typing; MRSA: meticillin-resistant *S. aureus*; NA: not applicable; ND: no data; R: routinely; ST: sequence type.

^a^ All samples includes both clinical and screening samples.

^b^ Sample collection is non-representative and/or non-systematic.

^c^ Catchment population: 1.0 million.

^d^ Catchment population: 1.2 million.

^e^ 11 of 14 isolates were a human clade of ST398.

^f^ Staphylococcal cassette chromosome *mec* (SCC*mec*) typing by *ccr* and *mec* complex determination.

^g^ PCR test for CC398 (*sau1-hsdS1* gene) [31].

^h^ *S. aureus* Genotyping Kit 2.0 (Alere Technologies GmbH, Jena, Germany).

^i^ Phenotyping using disk diffusion with a panel of antimicrobial agents and heavy metals along with an urease test.

^j^ Multilocus variable-number tandem repeat analysis (MLVA) [32], not specified whether routine or on indication.

^k^ Whole genome sequencing (WGS) during outbreak investigations.

^l^ Accessory gene regulator (*agr*) typing [33].

^m^ Potential LA-MRSA isolates of particular interest are subtyped by techniques including *scn/tetM/teK* PCR, microarray (Alere Technologies GmbH, Jena, Germany), SCCmec typing, MLST and/or in-house WGS.

### MRSA typing method

**Figure 2:**
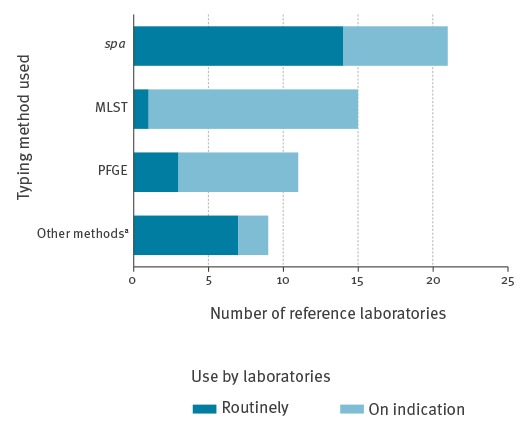
Availability of MRSA typing methods in 22 national/regional reference laboratories, 2013 (n = 21 European Union/European Economic Area countries)

MLST: multilocus sequence typing; MRSA: meticillin-resistant *Staphylococcus aureus.*

^a^ Other methods included: Staphylococcal cassette chromosome *mec* (SCC*mec*) typing by *ccr* and *mec* complex determination; PCR test for CC398 (*sau1-hsdS1* gene) [31]; *Staphylococcus aureus* Genotyping Kit 2.0 (Alere Technologies GmbH, Jena, Germany); phenotyping using disk diffusion with a panel of antimicrobial agents and heavy metals along with an urease test; multilocus variable-number tandem repeat analysis (MLVA) [32]; accessory gene regulator (*agr*) typing [33]; *scn/tetM/teK* PCR; and whole genome sequencing.

*Spa*-typing was the most widely used MRSA typing method in the responding reference laboratories (n = 21), followed by MLST (n = 15), PFGE (n = 11) and ‘other’ (n = 9) (Table 1). The majority of these laboratories had *spa*-typing available for routine use (n = 14/21), while MLST was only used routinely in Portugal (Figure 2).

### Typing of MRSA isolates

Overall, respondents reported receiving MRSA isolates from 14,291 patients in 2013, of which 13,756 (96.3%) were typed (Table 1).

**Figure 3:**
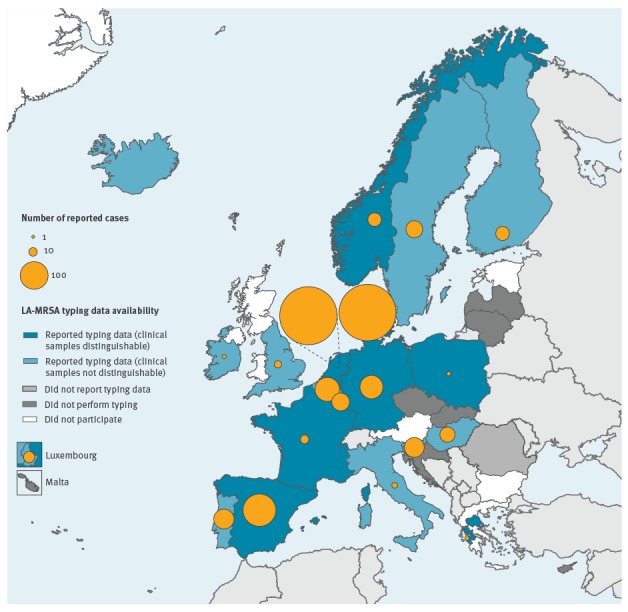
Number of LA-MRSA isolates reported to 21 national/regional reference laboratories in 20 European Union/European Economic Area countries, 2013 (n = 535)

LA-MRSA, both ST398 and other types, was identified by 17 of 19 countries with typing data (i.e. all participating countries that performed subtyping in 2013 except for Iceland and Portugal). The Netherlands, Denmark and Spain reported the largest numbers of LA-MRSA isolates (n = 164, 157 and 52, respectively; Table 1, Figure 3).

Administrative boundaries from EuroGeographics. Map produced on 20 October 2017.

LA-MRSA: livestock-associated meticillin-resistant *Staphylococcus aureus*; MRSA: meticillin-resistant *S. aureus.*

Overall, the percentage of typed MRSA isolates that were LA-MRSA was 3.9% (n = 535/13,756). For the nine NRLs that reported data from clinical samples only (i.e. excluding screening samples), this percentage was 9.0% (n = 417/4,612). In five NRLs (Belgium, Denmark, Spain, the Netherlands and Slovenia), LA-MRSA represented ≥ 10% typed MRSA isolates (Table 1).

Sweden also assessed the PVL status of its 14 MRSA that had an ST398-surrogate *spa* type and three of these were PVL-positive (all *spa* type t034), such that they were therefore not considered to be livestock-associated. England reported that 11 of the 14 MRSA CC398 identified belonged to the ‘human-adapted’ rather than ‘livestock-adapted’ clade as they were positive for PVL and/or immune evasion cluster-positive and/or tetracycline susceptible. This assignment was confirmed through WGS (personal communication, Angela M Kearns, January 2015). Poland assessed that the detected MRSA ST398 isolate was *spa* type t034 and PVL-negative, belonging to a ‘livestock-adapted’ clade that was immune evasion cluster-positive and tetracycline-resistant (personal communication, Joanna Empel, May 2016).

### Other LA-MRSAs

Almost all LA-MRSA isolates were CC398 (n = 533/535, 99.7%). Ten countries explicitly reported zero ‘other LA-MRSA’. Only Italy specified the non-CC398 subtypes that they considered to be LA-MRSA, i.e. two ST1 (t127) isolates from paediatric nasal screening samples.

### Type of sample/body site of microbiological samples

**Figure 4:**
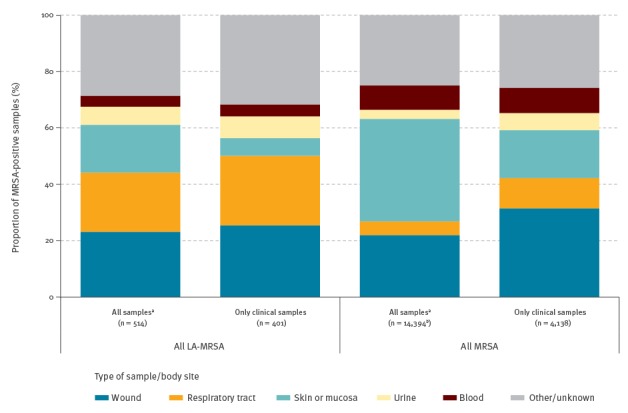
Type of sample/body site of MRSA-positive samples reported by 21 national/regional reference laboratories, 2013 (n = 20 European Union/European Economic Area countries)

LA-MRSA: livestock-associated meticillin-resistant *Staphylococcus aureus*; MRSA: meticillin-resistant *S. aureus*.

^a^ All samples includes both clinical and screening samples.

^b^ All data are included from isolates received by participating laboratories and subsequently reported in this survey. As Spain and Slovenia did not provide data on the total number of isolates received at the National Reference Laboratory in 2013 (see Table 1), the total number of isolates in this figure (n = 14,394) exceeds the total in Table 1 (n = 14,291).

The most common type of sample/body site for LA-MRSA-positive clinical samples was ‘other’, i.e. isolates from body sites/tissues other than blood, wound, urine, the respiratory tract, skin, mucosa and unknown data (n = 127/401, 32%). Of these 127 isolates, 117 were reported by Denmark. The next most common type of sample/body site for positive clinical samples were wound and respiratory tract, both 25% (Table 2, Figure 4).

**Table 2:** Type of sample/body site of MRSA-positive samples reported by 21 national/regional reference laboratories, 2013 (n = 20 European Union/European Economic Area countries)

Type of sample/body site of sample	All samples^a^ (n = 21 laboratories)				p value^b^	Only clinical samples (n = 9 laboratories)				p value^b^
	LA-MRSA (ST398)		non-LA-MRSA			LA-MRSA (ST398)		non-LA-MRSA		
	n	%	n	%		n	%	n	%	
Blood	20	4	1,225	9	< 0.001	17	4	355	9	< 0.001
Respiratory tract	108	21	590	4	< 0.001	99	25	345	9	< 0.001
Skin or mucosa	87	17	5,146	37	< 0.001	25	6	678	18	< 0.001
Urine	33	6	429	3	< 0.001	31	8	221	6	0.15
Wound	119	23	3,047	22	0.52	102	25	1200	32	0.006
Other/unknown	147	29	3,443	25	0.051	127	32	938	25	0.004
Total^c^	514	100	13,880	100	NA	401	100	3,737	100	NA

NA: not applicable; LA-MRSA: livestock-associated meticillin-resistant *Staphylococcus aureus*; ST: sequence type.

^a^ All samples includes both clinical and screening samples.

^b^ Chi-squared test compared detection of LA-MRSA vs non-LA-MRSA within each sub-category of isolation site.

**^c^** All data are included from isolates received by participating laboratories and subsequently reported in this survey. As Spain and Slovenia did not provide data on the total number of isolates received at the National Reference Laboratory in 2013 (see Table 1), the total number of isolates in this table (n = 14,394, all samples) exceeds the total in Table 1 (n = 14,291, all samples).

Overall, a greater proportion of LA-MRSA were isolated (p < 0.001) from clinical respiratory tract samples (n = 99/401, 25%) than were non-LA-MRSA (n = 345/3,737, 9%). Eighty-seven (88%) of these 99 LA-MRSA from clinical respiratory tract samples were reported by Denmark and the Netherlands. Inversely, a smaller proportion of LA-MRSA from all samples were isolated (p < 0.001) from blood samples (n = 20/514, 4%) than were non-LA-MRSA (n = 1,225/13 880, 9%) (Table 2). When considering only clinical samples, a smaller proportion of LA-MRSA were isolated (p < 0.001) from skin or mucosa samples (n = 25/401, 6%) than were non-LA-MRSA (n = 678/3,737, 18%) (Table 2).

## Discussion

The high response rate in the survey is indicative of the perceived public health importance of LA-MRSA for EU/EEA countries. In a similar survey of 2007 data, only seven of 15 European countries reported LA-MRSA isolates. In the current survey of 2013 data, LA-MRSA isolates were reported in all but two of the 20 countries that had MRSA typing data. Moreover, seven of 11 countries that participated in both surveys identified an increase in the proportion of MRSA that were LA-MRSA (CC398) [16]. Notably, in 2013 five geographically-dispersed European NRLs reported that more than one in 10 typed MRSA isolates were LA-MRSA. In Iceland (population 320,000), LA-MRSA was reported in either in the 2007 nor the 2013 survey, although two cases of MRSA CC398 were subsequently reported in 2014 [16,19].

CC398 remained the dominant LA-MRSA lineage of public health concern. Indeed, only one country listed another lineage that they considered to be LA-MRSA, ST1. Although the survey invited national respondents to use their considered expert opinion to specify non-ST398 lineages of LA-MRSA, it is possible that some non-ST398 LA-MRSA were misclassified as being non-LA-MRSA in some countries.

At least three countries performed additional laboratory tests to differentiate between ‘human-adapted’ and ‘livestock-adapted’ CC398 clades, e.g. by testing for PVL, presence of the immune evasion complex, tetracycline susceptibility and by WGS, all of which are also used by public health laboratories in the United States [5,24,25]. Detection of single-nt polymorphisms (SNPs) for *scn* and *tet(M)* genes, by WGS for example, is also appropriate to differentiate between human-associated and livestock-associated clades, respectively [26].

Given the apparent upward trend and more widespread dispersion of MRSA CC398 in Europe, the absence of MRSA typing in NRLs in seven countries in 2013 is of concern. Of these seven countries, the Czech Republic and Latvia (n = 20 and n = 8 laboratories, respectively) did provide aggregated *spa* type data in a 2011 subtyping pilot study in 26 European countries [18]. The Czech respondent confirmed that MLST and *spa*-typing were performed on MRSA isolates from blood samples up until 2011, while Latvia was considering including MRSA typing in their updated national AMR action plan and Cyprus had no plan to initiate typing. In 2014, Croatia's NRL received 140 MRSA isolates and all were characterised by *spa*, PVL, SCC*mec* typing and phenotypic susceptibility testing. None of the *spa* types were ST398 surrogates.

As in other surveys, LA-MRSA isolates were less likely to originate from blood than other MRSA isolates [13,16]. Fewer clinical samples skin or mucosa samples were positive for MRSA than ‘all samples’ as the latter included screening samples. The frequent detection of MRSA from respiratory tract samples relates to its prevalence in nares and the common use of this body site for screening.

More than half the countries that responded provided data on ‘all samples’ rather than solely clinical samples. It is suspected that the contribution of screening samples to the collated data are likely to have been sizable given that in 2013, clinical isolates comprised 157 of 643 (24%) MRSA ST398 in Denmark and 10 of 47 (21%) MRSA ST398 reported by Norway, with the remainder being screening samples (personal communication, Kjersti W Larssen and Petter Elstrøm, May 2016), [19,27].

The current study did not aim to provide information on the contribution of local and regional laboratories to national typing, although the contribution of the latter may have been notable in some European countries. For example, the Italian NRL provided typing data for all MRSA isolates including screening samples, none of which were ST398. This contrasts with a multidisciplinary investigation conducted in 2012–2013 in an Italian region where MRSA ST398 was identified from screening samples from rabbit holdings, including the holding’s workers and their relatives [28]. Systematic characterisation in 2010–2011 of all MRSA strains in a hospital in Lombardy, the region of Italy with the highest density of pig farming, identified MRSA ST398 in 5 of 879 nasal swabs and an isolate from otitis externa [29]. Spain’s NRL reported 52 LA-MRSA isolates in this current survey, explicitly stating that isolate ascertainment was neither nationally comprehensive nor systematic. In 2012–2013, a hospital in Catalonia reported 11 LA-MRSA ST398 [30]. Germany’s NRL reported 28 MRSA CC398 from clinical samples in 2013, while the University Hospital Münster, which has a catchment area with a high pig density in north-western Germany bordering the Netherlands, identified 267 MRSA ST398 cases in 2013, including 13 from clinical samples. Systematic screening of patients for MRSA carriage at admission to this hospital showed an increasing proportion of LA-MRSA carriers, from a first detection in 2000 to 19% and 35% of MRSA-positive screening samples in 2007 and 2013, respectively [31]. A case–control study of this hospital’s patients in 2013–2014 identified that 62% of MRSA CC398 cases reported direct livestock contact [32]. In 2014, this hospital detected MRSA ST398 in 202 admitted patient’s screening samples [31].

Since this survey of 2013 data, additional reports have indicated a continuing spread of LA-MRSA across Europe. The ‘Nordic MRSA’ database [15] identified the first detection of MRSA ST398 in Iceland in 2014 (n = 2) and an increase in MRSA ST398 detections between 2013 and 2014 in Denmark (from n = 643 to n = 1,276) and in Finland (n = 4 to n = 16). It also identified a decrease in Norway (n = 47 to n = 27). Not all MRSA ST398 in this database were livestock-associated (LA-MRSA). For example, the NRL in Sweden considered that only 10 of 13 MRSA ST398 in 2013 and 20 of 24 MRSA ST398 in 2014 were LA-MRSA, as they were also PVL-negative [19].

In Denmark, 43% of 2,965 human MRSA isolates (screening and clinical samples) in 2014 were ST398, with 89% of individuals reporting direct contact with pigs or being secondary contacts [33]. In Germany, a national multidisciplinary survey conducted between 2012 and 2015 in 17 equine hospitals and 39 veterinary practices identified MRSA ST398 in nasal swabs from 82.7% of 272 equine isolates and 19.2% of 349 individuals working with horses. Most isolates shared the same strain-type that was rarely found in the human databases of the NRL in Germany, implying direct transmission [34]. At a show-jumping event in Luxembourg in 2014, clones of MRSA ST398, confirmed by WGS, were detected in food and in throat swabs from catering staff [35]. In Sweden in 2014, a national survey of nucleus and multiplier pigs did not identify MRSA while MRSA ST398 was identified in horses and in 21 human cases [36].

Public health surveillance in Denmark and the Netherlands has detected LA-MRSA transmission without livestock contact with increasingly frequency [37,38]. Using next-generation sequencing to characterise MRSA isolates from 2003 to 2014, the Netherlands identified distinct lineages of LA-MRSA becoming more human-adapted, disseminating into the community [38]. In Denmark, a nationwide, retrospective temporo-spatial analysis of samples from 1999 to 2011 also showed that MRSA ST398 was capable of onward transmission in the community. The majority of individuals in the later years of the analysis had no livestock contact but were often clustered around those with livestock contact. There was little sign of substantial spread of MRSA ST398 in urban areas [33,37]. In February 2015, the detection of MRSA ST398 in two pre-packaged, processed pork products purchased from UK supermarkets and marked as UK farm origin attracted considerable media attention [39]. These findings, along with sporadic reports of MRSA CC398 from livestock, show that LA-MRSA is being increasingly found in the UK [40].

As was known at the outset, the results of this present study are not generalisable to European clinical laboratories, so the relative numbers of LA-MRSA identified by responding laboratories are not directly comparable and do not necessarily represent the national LA-MRSA prevalence. Sweden and England reported almost half of the typed MRSA isolates in this study; therefore, their surveillance data are over-represented. In Europe, there is considerable variability in sampling, criteria for referral to NRLs and typing strategies between countries [18]. Indeed, several participating NRLs emphasised that referrals for typing were neither systematic nor nationally representative. For example, the proportion of typed isolates that were associated with outbreak investigations and/or taken for clinical diagnostic purposes is unclear. Furthermore, the absence of reliably definable catchment areas for all laboratories hampers useful estimation of national or European LA-MRSA burden.

Linkage of clinical to microbiological and epidemiological data is not currently feasible in all European countries, removing the possibility of measuring the epidemiological association of cases with livestock [18]. Indeed, the majority of responding NRLs did not have data to differentiate clinical from total isolates. We therefore stratified analyses to compare cases with similar clinical impact. Additionally, because not all responding NRLs reported using subtype analysis to differentiate LA-MRSA within MRSA ST398, our study of ST398 may have overestimated the LA-MRSA burden.

The decision to omit a specific definition of ‘other LA-MRSA’ from the questionnaire was intended to provide the responding national experts with the opportunity to interpret ‘other’ broadly. No NRLs reported detection of ST9 or ST72, which are regularly detected in Asia, or other well-recognised LA-MRSA subtypes, such as ST5 [8-10]. Our approach may have kept respondents from stating more contentious interpretations of ‘livestock-associated’, resulting in an underestimation of the burden of less common non-ST398 LA-MRSAs.

In North America, livestock-associated meticillin-sensitive *S. aureus* (LA-MSSA) are more relevant for public health than LA-MRSA, including strains that are ST398 [5,24,25]. However, collection of MSSA data were outside the scope of the present study and may be worthy subject for a similar survey in Europe.

## Conclusions

The high response rate for this survey is indicative of the perceived public health importance of LA-MRSA in EU/EEA countries. The selection of a surveillance period before the increased media attention in Nordic countries in 2014 hopefully contributed to the comparability of the European data in the present study. This survey documents the increasing detection and geographical dispersion of LA-MRSA in humans in the EU/EEA since 2007, and highlights the public health and veterinary importance of LA-MRSA as a One Health issue. In the light of the increasing spread of LA-MRSA in Europe shown herein, we advocate that EU/EEA countries consider periodically repeating this survey to monitor changes. It is also suggested that isolates from veterinary sources be included in such monitoring to systematically map potential reservoirs and transmission pathways, thereby informing measures for prevention and control. Wherever practicable, attempts should be made to differentiate between ‘human-adapted’ and ‘livestock-adapted’ clades of MRSA CC398. European countries without this capability could consider other options, including cross-border collaborations, to characterise their MRSA isolates. We would also encourage linkage of multi-sectorial, One Health MRSA data to improve understanding of transmission pathways as well as to enable appropriate targeting and monitoring of the effectiveness of control measures.

## Notes

**European human LA-MRSA study group:** Belgium – Olivier Denis (Université Libre de Bruxelles, Brussels) Croatia – Ana Budimir (University Hospital Centre, Zagreb; School of Medicine, University of Zagreb, Zagreb) Cyprus – Despo Pieridou (Nicosia General Hospital, Nicosia) Czech Republic – Helena Žemličková (National Institute of Public Health, Prague) Finland – Laura Lindholm (National Institute for Health and Welfare, Turku) Germany – Franziska Layer (Robert Koch Institute, Berlin) Greece – Iris Spiliopoulou (National Staphylococcal Reference Laboratory, Department of Microbiology, University of Patras, Patras), Efthymia Petinaki (Department of Microbiology, University Hospital of Larissa, Larissa) Hungary – Ákos Tóth (National Center for Epidemiology, Budapest; European Programme for Public Health Microbiology (EUPHEM), European Centre for Disease Prevention and Control (ECDC), Stockholm, Sweden) Iceland – Gunnsteinn Haraldsson (Department of Clinical Microbiology, Landspitali University Hospital and Faculty of Medicine, University of Iceland, Reykjavik) Ireland – Gráinne I Brennan (National MRSA Reference Laboratory, St. James’s Hospital, Dublin) Italy – Monica Monaco (National Institute of Health, Rome) Latvia – Nicole Werner, Arta Olga Balode (Centre for Disease Prevention and Control, Riga) Lithuania – Jolanta Miciuleviciene (National Public Health Surveillance Laboratory, Vilnius) Luxembourg – Joël Mossong, Monique Perrin (Laboratoire National de Santé, Dudelange) Malta – Michael A Borg (Mater Dei Hospital, Valletta) Norway – Kjersti Wik Larssen (Norwegian MRSA reference laboratory, Trondheim), Petter Elstrøm (Norwegian Institute of Public Health, Oslo) Poland – Joanna Empel (Department of Epidemiology and Clinical Microbiology, National Medicines Institute, Warsaw) Portugal – Manuela Caniça, Vera Manageiro (Department of Infectious Diseases, National Institute of Health Doutor Ricardo Jorge, Lisbon) Romania – Irina Codita (National Institute of Research-Development for Microbiology and Immunology and University of Medicine and Pharmacy, Bucharest) Slovakia – Milan Nikš (Public Health Authority of the Slovak Republic, Bratislava) Slovenia – Urška Dermota (Department of Medical Microbiology, National laboratory for Health, Environment and Food, Maribor) Spain – María Dolores Pérez Vázquez, Ana Vindel (Institute of Health Carlos III, Madrid) Sweden – Sara Hæggman, Barbro Mäkitalo (Public Health Agency of Sweden, Solna) United Kingdom – Angela M Kearns, Bruno Pichon (Antimicrobial Resistance and Healthcare Associated Infections Reference Unit, Public Health England, London)
